# Physical activity, exercise, and mental disorders: it is time to move on

**DOI:** 10.47626/2237-6089-2021-0237

**Published:** 2021-10-22

**Authors:** Felipe Barreto Schuch, Davy Vancampfort

**Affiliations:** 1 Departamento de Métodos e Técnicas Desportivas Universidade Federal de Santa Maria Santa Maria RS Brazil Departamento de Métodos e Técnicas Desportivas, Universidade Federal de Santa Maria (UFSM), Santa Maria, RS, Brazil.; 2 Department of Rehabilitation Sciences Katholieke Universiteit Leuven Leuven Belgium Department of Rehabilitation Sciences, Katholieke Universiteit Leuven, Leuven, Belgium.

**Keywords:** Exercise, physical activity, mental disorders, physical health, mental health

## Abstract

**Introduction:**

Physical activity, conceptualized as any bodily movement that results in energy expenditure, and its structured form, exercise, play an important role in public health, preventing and treating a wide range of physical conditions, including metabolic and cardiovascular diseases and obesity.

**Objective:**

This article aims to provide a brief overview and summary of the evidence on: 1) the preventive effects of physical activity on a wide range of mental disorders; 2) the role of physical activity in promoting the physical health of people with mental disorders; 3) the role of exercise as a strategy to manage mental health symptoms in a range of mental disorders; and 4) the challenges and barriers faced when implementing exercise in clinical practice.

**Methods:**

This was a narrative review.

**Results:**

Compelling evidence has demonstrated that physical activity and exercise can also prevent common mental disorders, such as depression and anxiety disorders, and have multiple beneficial effects on the physical and mental health of people with a wide range of mental disorders. This body of evidence has been incorporated in national and international guidelines over the last decades, which have recommended the inclusion of physical activity and exercise as therapeutic approaches for mental disorders, mainly for depression and schizophrenia. Nonetheless, implementation into clinical practice has been slow, probably due to barriers associated both with patients and mental health professionals.

**Conclusion:**

Increases in physical activity levels in populations are likely to reduce the mental health burden. Exercise interventions should be incorporated to the routine care of people with mental disorders due its multiple benefits on physical and mental health outcomes. A multidisciplinary approach is needed to overcome patients barriers and enhance adherence and benefits.

## Introduction

Mental disorders are highly prevalent and burdensome. It is estimated that approximately 12% of the global population suffered from a mental disorder in 2019, accounting for roughly 5% of the disability-adjusted life years (DALYs) and 16% of the years lived with disability worldwide.^1^ The mental health burden is further compounded by a high prevalence of comorbid somatic disorders,^2-4^ leading to a life expectancy 15 to 20 years shorter when compared to that of the general population.^5^ Risk factors associated with this high rate of physical comorbidities are a genetic vulnerability in people with mental disorders, side effects of pharmacological treatments, and a poor lifestyle, including unhealthy eating habits, substance abuse, poor sleep, low levels of physical activity, and long periods spent on sedentary behavior.^6-9^

Expenses in mental health care, led by expenses in pharmacological interventions, have increased substantially over the last decades.^10^ However, current approaches have not been sufficient to further reduce the prevalence of these disorders at a population level – in fact, that prevalence has remained almost stable over the last 30 years worldwide. In fact, rates of common mental disorders such as depression and anxiety appear to even be increasing among the younger generations.^7^

Pharmacological treatments, including antidepressants^11^ and antipsychotics,^12^ are still the frontline strategy for symptom management. However, the long-term treatment effects of pharmacotherapy are being questioned.^13^ For many people with mental disorders, psychotropic medication does not result in clinically meaningful improvement in the long-term, and side effects such as significant weight gain,^12^ elevated blood glucose levels, and loss of sexual interest, among others,^14^ are considerable.^15,16^ These side effects often result in medication discontinuation and distress and can negatively impact patients’ lives.^17-19^ There has also been little improvement in the primary prevention of mental disorders, with clear gaps in both the evidence and implementation of such interventions.^6^

Thus, additional approaches towards the prevention and treatment of mental disorders, which can be delivered alongside or in the absence of traditional mental health care strategies, are needed to reduce the global and growing burden of these conditions. A large and new body of evidence has been accumulated over the last decades suggesting that physical activity and exercise might be such approaches. Although the body of evidence can be considered new, these interventions are not.

“Walking is man’s best medicine,” quoted Hippocrates (460-377 B.C.).^20^ The quote demonstrates that physical activity, nowadays defined as “any bodily movement produced by skeletal muscles that results in energy expenditure,”^21^ and exercise, defined as “a subset of physical activity that is planned, structured, and repetitive and has as a final or an intermediate objective the improvement or maintenance of physical fitness,”^21^ have been recognized, since the ancient Greeks of the classical period, as having preventative and therapeutic effects on human health. Currently, a plethora of evidence supports the claim that “exercise is medicine,”^22^ due to its preventative and therapeutic effects on a broad range of physical conditions, including neurological, metabolic, cardiovascular, and pulmonary diseases, musculoskeletal disorders, and cancer.^23^

A large body of evidence has demonstrated that these preventative and therapeutic effects are extended to mental health. This study aims to discuss the evidence available to date on: 1) the preventative effects of physical activity and exercise on mental disorders; 2) the role of physical activity and exercise in promoting the physical health of people with mental disorders; 3) the role of physical activity and exercise as strategies to manage mental health symptoms in a range of mental disorders; and 4) the challenges and barriers faced by mental health professionals when implementing exercise in clinical practice.

## Effects of physical activity on the prevention of mental disorders

Mental disorders are multifactorial and are linked to a multitude of nonmodifiable and modifiable risk and protective factors.^24-26^ There is available evidence from prospective and more complex Mendelian randomization studies that physical activity is a modifiable protective factor against the incidence of some mental disorders in people free from diagnosis.^27^

Evidence from a meta-analysis of 49 prospective studies, including data from more than 260,000 participants, demonstrates that people with higher physical activity levels are less likely to develop depression (odds ratio [OR] = 0.83, 95% confidence interval [95%CI] = 0.79-0.88).^28^ That relationship remained significant regardless of age group, sex, and geographic region and after adjustment for a number of potential confounders, including weight, smoking, and the presence of physical diseases or impairments.^28^ Even though the analysis was adjusted for multiple confounding factors, other behavioral, social, and genetic confounders cannot be fully ruled out in observational designs.^29^ Advanced epidemiological study designs, such as Mendelian randomization studies, can minimize these confounding factors and provide stronger insights on causal inferences.^29^ Mendelian randomization is an alternative method that uses the genetic variation of known genes associated with the outcome as a natural experiment, allowing the evaluation of a modifiable (nongenetic) exposure factor over the outcome in observational studies.^29^ Two Mendelian randomization studies reinforce the finding that physical activity protects from incident depression.^30,31^ First, a large-scale genome-wide association study using data from participants of the UK Biobank that assessed physical activity using objective measures (accelerometry) has shown that physical activity has causal protective effects against depression.^30^ The effect is consistent in magnitude with the findings of cohort studies (OR = 0.74, 95%CI = 0.59-0.92). This result is corroborated by a second, longitudinal, Mendelian randomization study using data from the Partners Biobank, a dataset collected from primary care patients. Interestingly, the authors found that physical activity can offset the risk of depression across all levels of genetic vulnerability to depression, including people at higher genetic risk.^31^

Evidence was also found for the protective effects of physical activity against incident stress and anxiety.^32^ A meta-analysis of 11 prospective studies, including more than 69,000 participants, demonstrated that higher levels of physical activity significantly reduced incident anxiety (OR = 0.74, 95%CI = 0.62-0.88) in the analysis adjusted for multiple confounding factors. The examination of specific anxiety disorders indicated protective effects against agoraphobia (OR = 0.43, 95%CI = 0.19-0.99) and post-traumatic stress disorder (OR = 0.58, 95%CI = 0.39-0.86).^32^ Because of the small number of studies on the topic, further exploration on the consistency of the effects across age groups, sex, and geographic regions was not possible.

There is limited and conflicting evidence on the protective effects of physical activity against bipolar disorders and schizophrenia. For bipolar disorder, one prospective study found that higher levels of physical activity were associated with a greater likelihood of incident bipolar disorder at follow-up.^33^ However, a Mendelian randomization study found that physical activity has a causal protective effect against bipolar disorders (OR = 0.49, 95%CI = 0.31-0.76).^34^ It is expected that additional studies will offer new insights into the topic.

Lastly, evidence from a meta-analysis of 5 prospective studies demonstrated that higher physical activity levels were associated with a lower risk of incident psychosis or schizophrenia (OR = 0.72, 95%CI = 0.53-0.99).^35^ However, when the analysis was limited to the two studies that adjusted for confounding factors, the significant associations were no longer supported.^35^ The Mendelian randomization study that addressed the topic does not support the notion that physical activity protects from psychosis or schizophrenia.^34^

## Effects of physical activity and exercise on the physical health of people with mental disorders

Physical activity is a well-established protective factor against diabetes, metabolic syndrome, cardiovascular diseases,^23^ and all-cause mortality in the general population.^36^ It was estimated, for example, that reducing the levels of physical inactivity by 10% worldwide would have resulted in more than 500,000 deaths averted in 2008.^37^

People with severe mental disorders, such as psychotic disorders, bipolar disorders, and depression, spend an average of 7.8 hours in sedentary behavior per day, being significantly more sedentary than age- and gender-matched controls.^9^ They also spend an average of 38 minutes per day in moderate to vigorous physical activity (95%CI = 32-45), significantly lower than controls. Moreover, they are 50% less likely to meet physical activity guidelines (OR = 1.5; 95%CI = 1.1-2.0) compared to controls.^9^ Among individuals with mental disorders, lower physical activity levels are associated with being male, being single, being unemployed, fewer years of education, higher body mass index (BMI), longer illness duration, antidepressant and antipsychotic medication use, and having a lower cardiorespiratory fitness. Notably, some differences were observed across diagnoses.^9^ People with schizophrenia were the least active, while people with bipolar disorder were the most physically active, although people with bipolar disorder are the ones reporting the higher levels of sedentary behavior.^9^

The literature has demonstrated that exercise programs result in reductions in body weight,^38^ waist circumference,^39^ BMI,^38^ epicardial adipose tissue,^38^ and subcutaneous adipose tissue^38^ in people with depression. Also, lifestyle counseling programs, including physical activity promotion and exercise programs, are the most effective interventions for weight reduction (standardized mean difference [SMD] = -0.98), followed by psychoeducation (SMD = -0.77), in people with schizophrenia.^40^

Changes in lipid profile, markers associated with incident cardiovascular diseases, and cardiovascular mortality are also evident.^41^ In people with depression, exercise significantly increases high-density lipoproteins levels.^38^ Lifestyle interventions have also proved effective in reducing triglycerides, total cholesterol, and low-density lipoprotein cholesterol (SMD ranging from -0.35 to -0.37) in people with schizophrenia.^40^

Lastly, exercise interventions yield large increases in exercise capacity in people with mental disorders,^42^ another independent risk factor for cardiovascular mortality. For example, a meta-analysis of randomized controlled trials (RCT) demonstrated that exercise interventions promoted increases equivalent to 3 mL/kg/min in people with depression^43^ and 2.79 mL/kg/min in people with schizophrenia.^44^ These increases in aerobic capacity are clinically meaningful, as they are associated with a decrement in all-cause and cardiovascular disease mortality.^45^ More specifically, increases of 3.5 mL/kg/min are associated with decrements of 13 and 15% in the risk of all-cause mortality and cardiovascular disease, respectively.^45^

## Effects of physical activity and exercise on the mental health symptoms of people with mental disorders

International guidelines for treating mental disorders have begun to incorporate the available scientific evidence and propose that physical activity and exercise should be integrated into mental health care.^46-49^ For example, the European Psychiatric Association’s guidelines on the promotion of physical activity in people with mental illness state that there is considerable empirical evidence supporting the use of physical activity interventions in treating major depressive disorder and schizophrenia spectrum disorders.^7^ Also, the Royal Australian and New Zealand College of Psychiatrists’ clinical practice guidelines for mood disorders propose that exercise should be incorporated as a routine care treatment even before pharmacotherapy or psychotherapy.^49^ The latest version of the Brazilian Medical Association’s guidelines on the treatment of depression, published back in 2009,^50^ does not mention the use of exercise or physical activity in the treatment of depression; however, exercise is listed in a separate guideline of nonpharmacological treatments.^46^ Currently, there is a plethora of evidence supporting the beneficial effects of physical activity and exercise in people with a range of mental disorders.^51^ These benefits are, for some diagnoses, extended beyond the core diagnostic symptoms, including improvements in cognitive symptoms and quality of life.^51^

Meta-analytical evidence demonstrates that exercise effectively reduces depressive symptoms in people with depression (people with subclinical symptoms or with a diagnosis of major depressive disorder).^52^ In people with major depression, the effect size was large (SMD = 1.13, 95%CI = 0.46-1.81) when compared to physically inactive control groups.^52^ In another meta-analysis, aerobic and mixed (aerobic and strength combined) exercises seemed to be more effective than strength exercises alone.^53^ However, a more recent review including a greater number of trials has demonstrated that strength training alone effectively reduces depressive symptoms, with a moderate effect size (Hedge’s *g* = 0.66, 95%CI = 0.48-0.83).^53^ Age and sex do not seem to alter the magnitude of the effect, suggesting that strength training is effective for all age groups and sexes. For depression, there is also evidence that exercise improves some cognitive outcomes, such as visual learning and memory (Hedge’s *g* = 0.24, 95%CI = 0.00-0.47),^54^ and improves physical (SMD = 0.53, 95%CI = 0.22-0.84) and psychological (SMD = 0.53, 95%CI = 0.22-0.85) domains of quality of life.^55^

Stubbs et al.^56^ conducted a meta-analysis including six trials assessing people with panic disorder (N = 2), generalized anxiety disorder (N = 1), or posttraumatic stress disorder (N = 2), in addition to a further RCT that included people with either panic disorder, social phobia, or generalized anxiety disorder.^56^ The authors found that exercise reduced anxiety symptoms with a moderate effect size (SMD = -0.58, 95%CI = -1.0 to -0.76). The small number of studies included precluded any further analyses exploring the effects of different types of exercise on specific diagnosis, or across age groups and sexes.^56^

In psychosis spectrum disorders and schizophrenia, a meta-analysis including 11 trials with at least 90 minutes of moderate to vigorous intensity exercising per week showed improvement of total symptoms (SMD = -0.72, 95%CI = -1.14 to -0.29), positive symptoms (SMD = -0.54, 95%CI = -0.95 to -0.13), and negative symptoms (SMD = -0.44, 95%CI = -0.78 to -0.09).^57^ In another study by the same authors, exercise also improved global cognition compared to non-physically active interventions of treatment-as-usual (Hedge’s *g* = 0.41, 95%CI = 0.19-0.64) in people with schizophrenia.^58^

There is some evidence from nonrandomized studies that exercise might effectively reduce depressive symptoms in people with bipolar disorders, but there is a paucity of studies reporting the effects of exercise on manic or hypomanic symptoms.^59^ In fact, some people with bipolar disorders refer to exercise as a “double-edged sword,” as it has the potential to be both beneficial and harmful at the same time, provoking relaxation and a lift in mood.^60^ Since these findings are based on qualitative studies, well-designed trials are required to clarify these effects in more detail.

Lastly, exercise programs have been shown to improve anxiety and depression in children with attention deficit hyperactivity disorder (weighted mean difference [WMD = -1.84, 95%CI = -2.65 to -1.03).^61^ Thought problems (WMD = -3.49, 95%CI = -5.51 to -1.47), social problems (WMD = -5.08, 95%CI = -7.34 to -2.82), and aggressive behaviors (WMD = -3.90, 95%CI = -7.10 to -0.70) are also improved following physical activity interventions.^61^ However, hyperactive/impulsive symptoms (WMD = -0.01, 95%CI = -0.32-0.29) and inattention symptoms (WMD = -0.22; 95%CI = -0.51-0.08) do not seem to improve significantly after exercise.^61^

## Barriers and challenges for the promotion of physical activity and exercise in people with mental disorders

Even though the evidence on the mental and physical health benefits of physical activity and exercise for people with mental disorders is growing fast, the translation of these beneficial findings into clinical practice is slow.^62^ For example, in a very recent survey evaluating 73 mental health professionals working on psychosocial rehabilitation centers in the metropolitan area of Porto Alegre, southern Brazil, including psychiatrists, general practitioners, psychologists, nurses, social workers, and exercise professionals (e.g., physiotherapists and physical educators), more than 40% never, and almost 22% occasionally prescribed or recommended exercise to their patients.^62^ Approximately 35% of the study participants usually or always prescribed or recommended exercise to their patients.^62^ In this scenario, determining the most common barriers to implementing exercise in prevention and treatment programs for people with mental disorders would help to optimize resource allocation when delivering physical activity and exercise services in clinical practice. Barriers are known to be multidimensional and include individual, patient-level and systemic, socio-ecological factors.^62^

In the previously mentioned study,^62^ the main reason for not prescribing or recommending exercise was rather systemic. Mental health professionals believe that exercise prescription is the responsibility of exercise professionals, such as exercise physiologists or physical therapists, and not a part of their own mental health profession.^62^ This is not surprising since exercise prescription is indeed considered a task for physical educators and physiotherapists within many health care systems, including in Brazil.^63^ However, there is emerging evidence that a multidisciplinary approach is necessary in order to increase physical activity and exercise adoption and maintenance. In other words, recommending, discussing, and proposing lifestyle changes, while at the same time exploring individual barriers to such changes, should be a result of combined, coordinated efforts of all professionals involved in patient care, and not only of exercise professionals.^64^ For example, receiving a recommendation from a physician and other health care professionals is associated with greater exercise engagement in people with anxiety and depression.^65^ Therefore, in order to move on, physical activity and exercise should be considered everyone’s responsibility in multidisciplinary prevention and treatment programs for people with mental disorders.

Patient-level barriers can be physical or psychological. The most common physical barriers are poor physical health and fatigue. In the psychological arena, high levels of stress and depression, as well as a lack of motivation, have been reported.^66^ To help patients overcome these personal barriers, evidence-based theory-driven cognitive behavioral strategies that facilitate autonomous motivation have been shown to be effective^67^ and should be adopted. Autonomous motivation includes for example having identified motives for exercising, i.e., experiencing the personal importance of exercise due to physical, mental, or social benefits (identified regulation). Another related form of autonomous motivation is integrated regulation, in which motives for exercising are brought in harmony with other prevailing life values, e.g., being an active contributor to the community, for which one needs to be healthy – to the point that being active becomes prioritized within several life domains. A third form of autonomous motivation includes intrinsic regulation, which entails engaging in exercise for its own sake, i.e., because one finds exercising stimulating, challenging, or enjoyable by itself. Autonomous motivation differs from controlled regulations such as exercising to avoid criticism or to obtain external appreciation (external regulation). The imposition of pressures onto one’s own functioning, for instance, by reinforcing one’s exercise engagement with feelings of guilt, self-criticism, or contingent self-worth, is also a controlled form of exercise motivation, i.e., a person could experience feelings of guilt, shame, or low self-worth when not exercising (introjected regulation). Therefore, instead of engaging in exercise for the experienced benefits, importance, or enjoyment, they engage for thinking that others would consider them lazy otherwise.^67-69^

Previous research has shown that to facilitate autonomous motivation, psychological needs for autonomy (i.e., experiencing a sense of psychological freedom when exercising), competence (i.e., feeling effective to attain desired outcomes), and relatedness (i.e., being socially connected) need to be nurtured.^70^ In clinical practice, this can be achieved by offering clear exercise choices, supporting the patient’s own initiative, avoiding the use of external rewards, using autonomy supportive language (e.g., “could” and “choose” rather than “you should exercise” and “you have to do aerobic exercise three times per week”), giving constructive feedback, showing enthusiasm and interest, and offering both individual support and group-based exercise sessions. Exploring individual preferences, views, and concerns is particularly important.^67-69^ Personal preferences should be considered in all and every aspect that involves exercise practice. For example, there is evidence that both aerobic^52,56^ and strength training^53,71^ effectively reduce depressive and anxiety symptoms, therefore patients should have the opportunity to choose the activity they prefer. Regarding intensity, self-selected intensities are more likely to be associated with positive affect in those with lower fitness and physical activity levels.^72^ Self-selected intensity also facilitates feelings of competence. Lastly, social relatedness is not only an important motivator but also a potential moderator for clinical improvement in exercise.^73^ Thus, encouraging patients to exercise with friends and family may enhance not only the implementation but also the outcome of the exercise program.^73^

Other important barriers at the systemic socio-ecological level include a lack of social support and a lack of access to appropriate exercise facilities, including high costs in private settings.^66^ Therefore, policy-makers and health service managers should work together to build a supportive environment for physical activity and exercise promotion by listening to practitioners and consumers’ wills and needs and seeking strategies to provide access to exercise facilities, in addition to including exercise professionals in mental health staff. Exercise is everyone’s responsibility, and all mental health professionals have an important role to play, including exercise professionals. The inclusion of exercise professionals in multidisciplinary mental health teams has been shown to be beneficial, too. Previous meta-analyses demonstrated that exercise interventions supervised by trained exercise professionals are more effective than nonsupervised interventions^6,57^ and have a lower risk of dropout.^74-76^

## Conclusions

The current evidence demonstrates that physical activity increments at a population level is likely to reduce the prevalence of mental disorders, particularly common mental disorders (depression and anxiety). From a clinical perspective, people with mental disorders are a vulnerable group, with physical health disparities. One of the reasons for these disparities can be attributed to poor lifestyle choices, such as low physical activity levels. Physical activity is a well-known protective factor against cardiometabolic diseases, and embedding it into routine care will very much likely help reduce this health disparity while also helping to keep mental health symptoms at bay. Incorporating physical activity and exercise programs is a multidisciplinary task that requires attention and effort from all mental health professionals working collaboratively. Although exercise professionals are the ones who prescribe and supervise exercise sessions, exploring patient-level barriers and developing strategies to overcome them is everyone’s responsibility. In this sense, self-determination theory provides evidence-based strategies that assist health professionals in helping patients to overcome their barriers. Efforts towards integrating physical activity and exercise interventions within routine mental health care should avoid focusing solely on individual-level behavioral changes and should include broader changes to service structure, delivery, and culture. Therefore, in order to move on and translate the scientific evidence available into clinical practice, all mental health professionals should receive basic training in the principles of physical activity prescription. In addition, experts in exercise prescription should receive adequate training on mental health care to be incorporated in multidisciplinary mental health settings.
